# Supplementary material for: Ebola Virus VP35 Interacts Non-Covalently with Ubiquitin Chains to Promote Viral Replication Creating New Therapeutic Opportunities
Source: bioRxiv. 2023 Jul 15:2023.07.14.549057. Preprint. [Version 1] doi: 10.1101/2023.07.14.549057 (PMC10369991; doi:10.1101/2023.07.14.549057)
Supplement: 1 [file NIHPP2023.07.14.549057V1-supplement-1.pdf]

bioRxiv preprint doi: https://doi.org/10.1101/2023.07.14.549057; this version posted July 15, 2023. The copyright holder for this preprint (which was not certified by peer review) is the author/funder, who has granted bioRxiv a license to display the preprint in perpetuity. It is made available under aCC-BY 4.0 International license.

SUPPLEMENTARY DATA

Ebola Virus VP35 Interacts Non-Covalently with Ubiquitin Chains to Promote Viral Replication Creating New Therapeutic Opportunities.

Carlos A. Rodríguez-Salazar [1,2,#], Sarah van Tol [1,#], Olivier Mailhot [3,#,‡], Gabriel Galdino [3,#], Natalia Teruel [3], Lihong Zhang [4], Abbey N. Warren [1,5], Maria Gonzalez-Orozco [1], Alexander N. Freiberg [4], Rafael J. Najmanovich [3,*], Maria I. Giraldo [1,*], Ricardo Rajsbaum [1,5,*].

1- Department of Microbiology and Immunology, University of Texas Medical Branch, Galveston 77555, Texas, USA.
2- Molecular Biology and Virology Laboratory, Faculty of Medicine and Health Sciences, Corporación Universitaria Empresarial Alexander von Humboldt, Armenia 630003, Colombia.
3- Department of Pharmacology and Physiology, Faculty of Medicine, Université de Montréal, Montreal, Canada
4- Department of Pathology, University of Texas Medical Branch, Galveston 77555, Texas, USA.
5- Center for Virus-Host-Innate Immunity and Department of Medicine; Rutgers Biomedical and Health Sciences, Institute for Infectious and Inflammatory Diseases, Rutgers University, Newark, New Jersey 07103.

# These authors contributed equally.
*Corresponding author name(s): Rafael Najmanovich, Maria Giraldo and Ricardo Rajsbaum.
‡ Current address: Department of Pharmaceutical Chemistry, University of California–San Francisco, San Francisco, CA, USA

Email: rafael.najmanovich@umontreal.ca,
migirald@utmb.edu,
ricardo.rajsbaum@rutgers.edu

Supplementary table 1 shows the predicted contributions of individual interactions in kcal/mol to the binding energy between VP35 and Ubiquitin calculated by gRINN and Surfaces. The first row in blue shows the residue ARG225, predicted as the highest and second highest contributing favorable interaction respectively by gRINN and Surfaces.

Supplementary table 2 shows the difference in predicted contributions of individual interactions to the ΔG of binding for different mutants in position 225. In blue in the first row the original ARG residue. Note that the value for the interaction with GLUE18 alone is the same as what is presented in Supplementary Table 1. The second value (overall), represents the sum of all interactions with the specific residue. For example, R225 makes additional favorable interactions with other residues that contribute to the total energy of binding. The values in salmon highlight the predictions for the mutations for K225 and E225.

bioRxiv preprint doi: https://doi.org/10.1101/2023.07.14.549057; this version posted July 15, 2023. The copyright holder for this preprint (which was not certified by peer review) is the author/funder, who has granted bioRxiv a license to display the preprint in perpetuity. It is made available under aCC-BY 4.0 International license.

Supplementary Table 1. Comparison of contribution to binding energy of gRINN and Surfaces predictions in kcal/mol of individual interactions within the VP35-Ub complex interface.

VP35	Ub	gRINN	Surfaces
ARG225	GLU18	-14.81	-1.11
LYS222	GLU16	-14.00	0.00
ARG305	ASP58	-10.60	-0.75
ARG298	GLU24	-10.43	-1.85
ASP230	LYS63	-9.00	0.00
TYR229	GLU18	-4.45	-0.59
GLN244	GLU18	-1.99	-0.01
PRO304	ASP58	-1.65	0.00
ARG305	SER57	-1.63	-0.99
LYS222	GLU18	-1.60	0.00

Supplementary Table 2. Predicted contributions of different residues modeled in position 225 to the binding energy in kcal/mol overall or specifically with GLU18.

Mutation	Contribution to Binding Energy	
	Overall	GLU18
ARG225	-1.27	-1.11
TRP225	-0.81	-0.81
LYS225	-0.57	-0.47
GLN225	-0.56	-0.56
THR225	-0.03	-0.03
ASN225	0.10	0.10
LEU225	-0.03	-0.03
VAL225	-0.05	-0.05
ILE225	-0.10	-0.10
MET225	-0.12	-0.12
CYS225	0.00	0.00
SER225	0.00	0.00
PRO225	0.00	0.00
GLY225	0.00	0.00
HIS225	0.00	0.00
ALA225	0.00	0.00
TYR225	0.00	0.00
GLU225	0.07	0.07
PHE225	0.00	0.00
ASP225	0.13	0.13
